# Plastocyanin and Cytochrome *f* Complex Structures Obtained by NMR, Molecular Dynamics, and AlphaFold 3 Methods Compared to Cryo-EM Data

**DOI:** 10.3390/ijms252011083

**Published:** 2024-10-15

**Authors:** Ilya Kovalenko, Vladimir Fedorov, Sergei Khruschev, Taras Antal, Galina Riznichenko, Andrey Rubin

**Affiliations:** 1 Faculty of Biology, Lomonosov Moscow State University, Moscow 119234, Russia; xbgth@ya.ru (V.F.); styx@biophys.msu.ru (S.K.); riznich@biophys.msu.ru (G.R.); rubin@biophys.msu.ru (A.R.); 2 Institute of Computer Science and Mathematical Modeling, Sechenov First Moscow State Medical University (Sechenov University), Moscow 119991, Russia; 3 Laboratory of Integrated Ecological Research, Pskov State University, Pskov 180000, Russia; taras_an@mail.ru

**Keywords:** protein–protein interaction, complex formation, Brownian dynamics, molecular dynamics, AlphaFold, NMR, plastocyanin, cytochrome *f*

## Abstract

Plastocyanin is a small mobile protein that facilitates electron transfer through the formation of short-lived protein–protein complexes with cytochrome *bf* and photosystem 1. Due to the transient nature of plastocyanin–cytochrome *f* complex, the lack of a long-lived tight complex makes it impossible to determine its structure by X-ray diffraction analysis. Up to today, a number of slightly different structures of such complexes have been obtained by experimental and computer methods. Now, artificial intelligence gives us the possibility to predict the structures of intermolecular complexes. In this study, we compare encounter and final complexes obtained by Brownian and molecular dynamics methods, as well as the structures predicted by AlphaFold 3, with NMR and cryo-EM data. Surprisingly, the best match for the plastocyanin electron density obtained by cryo-EM was demonstrated by an AlphaFold 3 structure. The orientation of plastocyanin in this structure almost completely coincides with its orientation obtained by molecular dynamics calculation, and, at the same time, it is different from the orientation of plastocyanin predicted on the basis of NMR data. This is even more unexpected given that only NMR structures for the plastocyanin-cytochrome *f* complex are available in the PDB database, which was used to train AlphaFold 3.

## 1. Introduction

**Figure 1:**
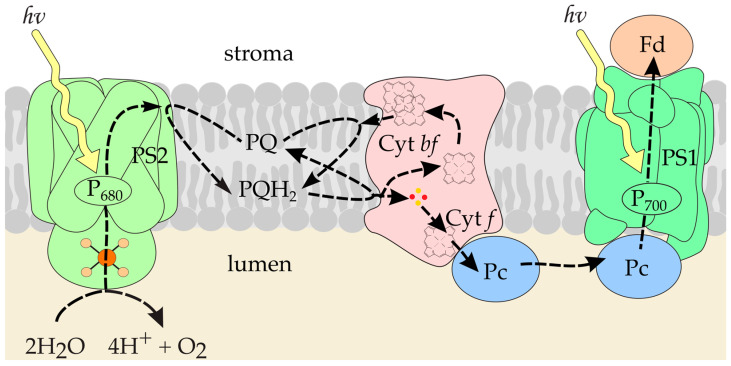
The scheme of electron transfer processes in a photosynthetic membrane. The diagram includes the components of the electron transfer chain: PS2, PS1, the photosystems 2 and 1; Cyt *bf,* cytochrome *bf* complex; PQ, plastoquinone pool; Fd, ferredoxin; Pc, plastocyanin.

Photosynthesis is the process that ensures the existence of life on Earth. This is the only process in living systems where energy is accumulated; all other processes occur with energy consumption. During the primary processes of photosynthesis, solar energy is converted into the energy of chemical bonds, and oxygen is released, which we breathe. The uniqueness of this process is associated with the light-induced electron transfer along the so-called “photosynthetic chain of carriers” (Figure 1), which results in photosynthetic phosphorylation and reduction of pyridine nucleotides, the products necessary for biosynthesis [1].

The main participants in the process of photosynthetic electron transport—multi-enzyme complexes of photosystem 2 (PS2), cytochrome complex (Cyt *bf*), and photosystem 1 (PS1)—are built into the bilayer lipid membrane and provide directed electron transfer through the photosynthetic membrane [2]. Communication between PS2 and Cyt *bf* is carried out by plastoquinone (PQ) molecules that diffuse inside the membrane. Intermediates between the Cyt *bf* and PS1 complexes are plastocyanin (Pc) protein molecules diffusing in the lumen. The Pc molecule binds to the subunit *f* (cytochrome *f*, Cyt *f*) of the Cyt *bf* complex, takes an electron, and transfers it to PSI complex via diffusion.

Pc facilitates electron transfer through the formation of short-lived protein–protein complexes with Cyt *f* and PS1 [3]. The formation of such transient complexes is required in order to maintain high-turnover conditions along the electron transfer chain [4]. An essential feature of transient protein interactions is fast dissociation, which ensures the limited lifetime of the complex [5]. The interactions formed in such protein−protein complexes are specific but highly dynamic [6]. Transient protein complexes, such as those formed by Pc and Cyt *f*, are thought to exist as an ensemble that can contain a large population of loosely bound states [7]. This population is in rapid exchange with one or more states that are presumably more optimal for electron transfer. The precise molecular mechanisms of Pc–Cyt *f* complex formation and the critical interactions involved in this process are still under question and, moreover, vary among species [6,8].

**Figure 2:**
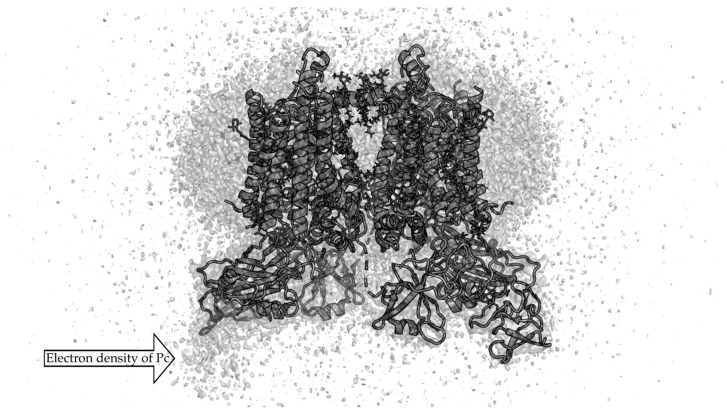
Secondary structure of Pc–Cyt *bf* complex (PDB id 7qrm [13]) superimposed on the electron density obtained by cryo-EM (EMDB entry EMD-14123 [13]). EMD-14123 density is depicted by grey isosurface 0.15 a.u.

Due to the transient nature of such a complex, the lack of a long-lived tight complex makes it impossible to determine its structure by X-ray diffraction analysis. It is convenient to study the structures of such complexes using NMR in solution [9], and, more recently, by cryo-EM methods [10]. The orientation of spinach [11] and poplar [12] Pc in complex with turnip Cyt *f* has been determined by rigid-body calculations using restraints from paramagnetic NMR measurements. In paper [13], an electron density map of the Cyt *bf* complex with bound Pc was obtained by cryo-EM (Figure 2). The hydrophobic parts of the Cyt *bf*, which are embedded in the thylakoid membrane, are surrounded by a mixture of ordered and disordered lipids and detergent molecules. The electron density corresponding to the Pc molecule is significantly weaker than that of the Cyt *bf* complex. This did not allow to distinguish the individual secondary structure elements of Pc, although the relative position of Pc in a Pc–Cyt *bf* complex is clear.

Computer methods are also widely used to predict Pc–Cyt *f* encounter and final complex structures. Rigid-body Brownian dynamics has been used many times to determine the structures of encounter complexes of these proteins [14,15,16,17]. A coarse-grained Langevin dynamics approach was also applied [18]. To simulate electron transport transient protein–protein complex formation, we suggested a two-step approach, where the binding process of two proteins can be divided into two principal stages [8]. The first of them is the encounter complex formation, where diffusion and long-range electrostatic interactions play the key role [19]. This first stage can be conveniently simulated by the rigid body Brownian dynamics (BD) method with explicit electrostatics [20], and the result of this simulation is the set of electrostatically favorable encounter complexes. At the second stage, hydrophobic and van der Waals interactions, as well as the mobility of atoms in the protein–protein interface, play a crucial role in the further approaching of macromolecules resulting in their final binding. This second stage is suitable to simulate using the explicit solvent full-atom molecular dynamics (MD) method.

**Figure 3:**
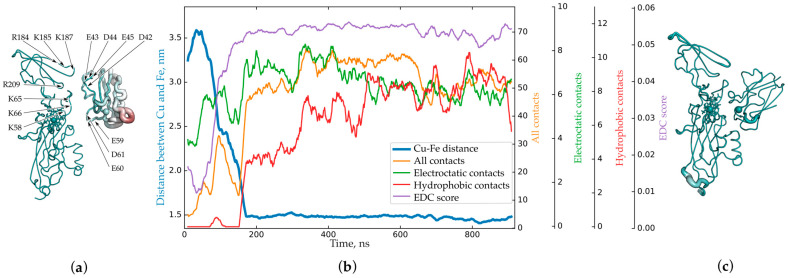
(**a**) Central structure of the first cluster of encounter complexes of Pc and Cyt *f* from higher plants with electrostatic energy of more than 8 kT [8]. (**b**) Dependence of the number of hydrophobic (red), electrostatic (green), and all (orange) contacts and EDC score (lilac) on time in comparison with the change in distance (blue) between iron and copper atoms obtained from MD calculations, based on data from [21] and current research (EDC score). (**c**) Structure of the final complex obtained from MD calculations. Structures are colored according to the value of the B-factor from emerald (0) to ruby, 7263 A^2^ in (**a**) and 2074 A^2^ in (**c**). The thickness of the lines of protein structures is proportional to the value of the B-factor. Panels (**a**,**c**) are reprinted from [8] with permission from John Wiley & Sons, Inc. © 2019 Scandinavian Plant Physiology Society.

The variety of structures in the set obtained by the BD method can be very large, and, therefore, they need to be analyzed in some way in order to select representative structures to start MD calculations, keeping in mind that each MD simulation takes a lot of computer time and power. For such an analysis, we adapted a hierarchical density-based clustering algorithm. We obtained Pc–Cyt *f* encounter electrostatic complexes and simulated their transformation into the final ones for higher plants, green algae and cyanobacteria [8]. For higher plants, in ‘productive’ electrostatic complexes (i.e., complexes which can evolve to functionally active ones, Figure 3a), the negatively charged loop D42E43D44E45 of Pc attracts both the positively charged region of Cyt *f*, consisting of the amino acid residues K58, K65 and K66, and the R184K185E186K187 loop. Additionally, another negatively charged loop, the E59E60 of Pc, attracts the positively charged residue K122 of Cyt *f* [8,21]. This interaction forms a flexible electrostatic joint of Pc with Cyt *f* with no hydrophobic contacts involved (Figure 3b).

During the formation of a functionally active complex (Figure 3c), electrostatic contacts between the same two pairs of oppositely charged regions of two proteins are preserved, and new ones are formed; that is, the total number of electrostatic contacts increases slightly (Figure 3b). As the distance between protein cofactors decreases, hydrophobic contacts appear. These contacts stabilize the resulting final complex with a Cu–Fe distance of less than 1.5 nm, due to which, the reaction centers of the proteins are brought closer together for a time sufficient for electron tunneling [21].

Thus, to date, a fairly large number of slightly different structures of the Pc–Cyt *f* complex have been obtained by experimental and computer methods. Recently, another method for predicting the structures of intermolecular complexes has become available. This is the artificial intelligence (AI) method, implemented, for instance, in the AlphaFold 3 tool created by Google DeepMind [22] and available since May, 2024. AlphaFold 3, unlike its previous version, AlphaFold 2, is designed to predict the spatial structures of intermolecular complexes and takes into account not only the protein part itself, but also various cofactors, such as metal ions, hemes, and chlorophylls.

In the present study, we compare encounter and final complexes obtained by the Brownian and molecular dynamics methods, as well as the structures predicted by AI, with NMR and cryo-EM data. The structures obtained by modern computational methods are in good agreement with experimental data. Surprisingly, the best match to the Pc electron density obtained by cryo-EM was demonstrated by a structure predicted by AlphaFold 3.

## 2. Results

In this study, we compare the position of a Pc molecule in a complex with a Cyt *f* molecule obtained by various methods with the spatial distribution of Pc electron density in a complex with Cyt *bf*. In cryo-EM microscopy, to assess the quality of the theoretically predicted structure, certain correlation coefficients are calculated, comparing experimental and predicted spatial distributions of electron density [23]. However, such correlation coefficients are too sensitive to the positions of individual atoms and are, therefore, not suitable for solving our problem of comparing the position of the protein molecule as a whole with the spatial distribution of electron density. In this work, in order to ensure that small changes in the spatial atom positions do not affect the assessment of the position of the protein as a whole, we smoothed out spatial fluctuations of the electron density by averaging the values in neighboring cells and used the resulting smoothed electron density values as a measure for assessing the correspondence between a certain atom position and its electron density. We use the smoothed electron density value averaged over Pc C_α_ atoms as a measure of the compliance of the considered Pc–Cyt *f* complex structure to the experimental electron density. We will further refer to this electron density compliance score as the EDC score.

**Figure 4:**
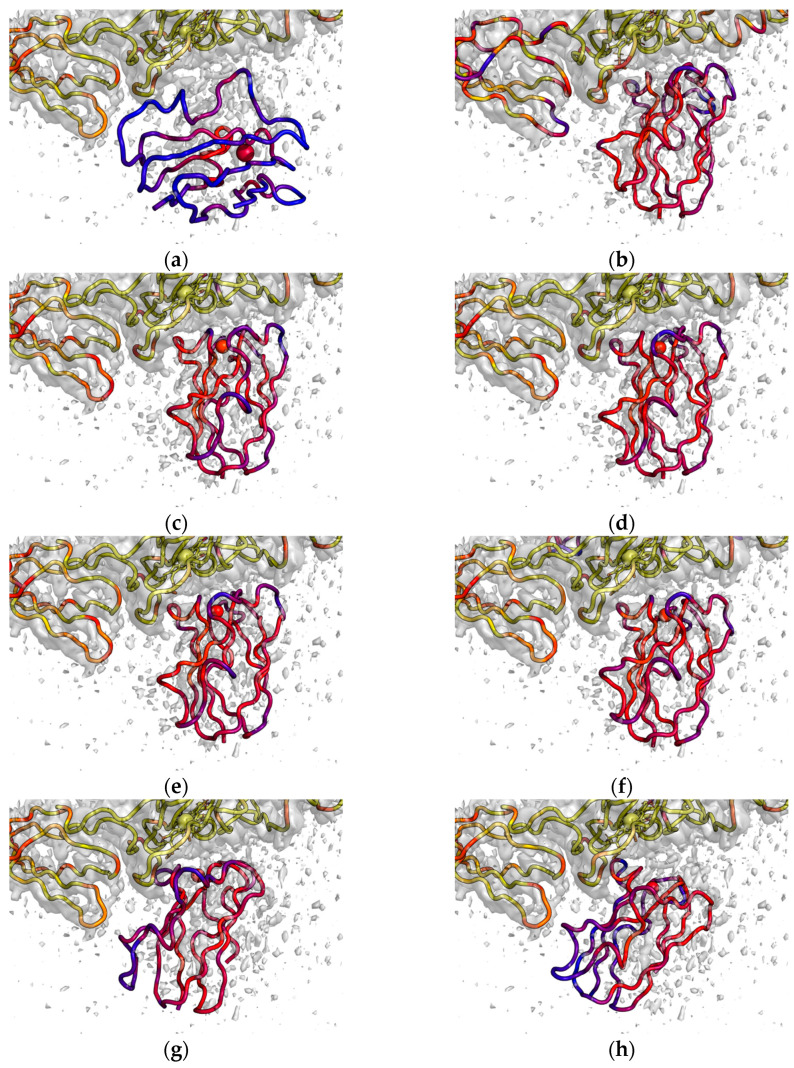
Backbone structure of Pc–Cyt *f* complexes aligned to Cyt *f* of Cyt *bf* complex (PDB id 7qrm [13]) superimposed on the electron density obtained by cryo-EM (EMDB entry EMD-14123 [13]). (**a**) Central structure of encounter complexes cluster obtained by BD for spinach Pc and turnip Cyt *f*. (**b**) Final complex obtained by all-atom MD, initiated from the structure in panel (**a**). (**c**) Complex of spinach Pc and turnip Cyt *f* predicted by AlphaFold 3 with the highest ipTM score. (**d**) Complex of spinach Pc and turnip Cyt *f* predicted by AlphaFold 3 with the lowest ipTM and the highest EDC scores. (**e**) Complex of spinach Pc and spinach Cyt *f* predicted by AlphaFold 3 with the lowest ipTM and the highest EDC scores. (**f**) Complex of spinach Pc and spinach Cyt *bf* predicted by AlphaFold 3 with the highest ipTM and the highest EDC scores. (**g**) NMR structure (PDB id 2pcf, model 9) for spinach Pc and turnip Cyt *f*. (**h**) NMR structure (PDB id 1tkw, model 7) for poplar Pc and turnip Cyt *f*. Backbone and cofactors of Pc and Cyt *f* molecules are colored by EDC score from blue (zero and lower) to yellow (0.15 and higher). EMD-14123 density is depicted by grey isosurface 0.15 a.u.

Figure 4 shows Pc molecules in complexes with Cyt *f* obtained by various methods and superimposed on the experimental electron density of the Pc–Cyt *bf* complex obtained by cryo-EM, EMDB entry EMD-14123 [13]. Table 1 presents the numerical comparison of these Pc–Cyt *f* complexes. To evaluate the differences in the Pc position and orientation in the eight structures shown in Figure 4, we calculated the distances between the center of mass of the Pc molecule (upper triangle cells with grey background in Table 1) and RMS distances between Pc C_α_ atoms (lower triangle cells in Table 1) for all these structures. To characterize individual structures, we calculated Cu–Fe distances and EDC scores (outlined diagonal values).

In Figure 4a, we can see that the central structure of the cluster obtained by the BD method for spinach Pc and turnip Cyt *f* poorly matches the EMD-14123 electron density, and the EDC score is low and equal to 0.026 (Table 1). This is not surprising, since BD takes into account electrostatic interactions only. The difference between structures in this cluster is quite large, with a maximum RMSD of 41 Å. In a subsequent MD simulation, the Pc molecule rotates to a position much better suited to the electron density, and at the same time, the distance between the copper and iron atoms is reduced from 28.4 Å to 14.3 Å and the EDC score increases up to 0.056 (Figure 3b). The difference between the final complex structures is lower, with a maximum RMSD of 9 Å. We calculated the central structure of the final complexes; this structure demonstrates a much higher EDC score value equal to 0.056 (Figure 4b, Table 1). The maximal RMSD of the final complex structures to this central structure is 6 Å while the mean RMSD is 2.3 Å.

In this study, we used the AlphaFold 3 tool to predict the structure of Pc–Cyt *f* complex. A set of five complex structures was obtained for spinach Pc and turnip Cyt *f*. The predicted template modeling (pTM) score varies from 0.84 to 0.85 and the interface predicted template modeling (ipTM) score varies from 0.82 to 0.84. These five structures are rather similar to each other, with a maximum RMSD of 4.5 Å. The structure with the highest ipTM score, 0.84 (Figure 4c, Table 1), has an EDC score equal to 0.055 and Cu–Fe distance 13.3 Å. However, the structure with the lowest ipTM score, 0.82, best suits the electron density demonstrating the highest EDC score, equal to 0.059, and the lowest Cu–Fe distance, 12.6 Å, among all five AlphaFold-predicted structures (Figure 4d, Table 1).

Despite sequence differences between spinach and poplar Pc (see Supplementary Files), the structures of the complexes predicted by AlphaFold 3 for spinach Pc–turnip Cyt *f* and poplar Pc–turnip Cyt *f* are very similar. This is not surprising because all amino acid replacements do not affect the protein–protein interface. The RMS distance between Pc C_α_ atoms in two structures with the highest ipTM score is 0.69 Å. Similarly, sequence differences between spinach and turnip Cyt *f* (see Supplementary Files) do not lead to significant differences in the AlphaFold-predicted Pc–Cyt *f* complex structure (Figure 4e, Table 1). The difference between predicted complexes of Pc with soluble part of Cyt *f* and the complete structure of Cyt *bf* is also insignificant and does not exceed the minimal predicted aligned error (PAE) values calculated by AlphaFold 3. Note that the PAE for Pc in a complex with Cyt *f* (3.5 Å) is three times lower than for Pc in a complex with Cyt *bf* (10.6 Å).

We calculated the EDC score for all 10 models of spinach Pc–turnip Cyt *f* complex (PDB ID 2pcf [11]) and all 10 models of poplar Pc–turnip Cyt *f* complex (PDB ID 1tkw [12]) determined by paramagnetic NMR and restrained rigid-body MD. The models with the highest EDC score values are presented in Figure 4g (2pcf, model 9) and Figure 4h (1tkw, model 7). These complexes have EDC scores equal to 0.052 and 0.046 (Table 1), which is noticeably less than scores for complexes obtained by combined BD+MD method and by AlphaFold 3. Note that Cu–Fe distance in model 9 of 2pcf is 11.4 Å, which is the greatest value among all 10 structures. On the contrary, model 8 has the lowest Cu–Fe distance equal to 10.5 Å, but its EDC score is as low as 0.046.

The central structure of the encounter complexes obtained by the BD method is most different from all other structures. The RMS distances between Pc C_α_ atoms in this particular structure and all the other structures exceed 19 Å (Table 1). The structures of the complex obtained by BD+MD and AlphaFold 3 do not differ much from each other: The RMS distance between the MD structure and the AlphaFold 3 structure, which best matches the electron density, is only 3.3 Å, while the center of mass is shifted by only 2.2 Å. Naturally, the best and worst Alphafold 3 structures differ even less from each other: The RMS is less than 2.4 Å, and the distance between centers of mass in these two structures is 1.9 Å. In turn, the NMR structures of the spinach and poplar plastocyanin complexes also differ from each other; the RMS distance between them is 6.6 Å, and the positions of the center of mass differ by 4.5 Å. Surprisingly, both the MD structure and the AlphaFold 3 structures are significantly different from the NMR structures. Indeed, the RMS distances between them are not less than 7.1 Å, and the positions of the center of mass differ by a minimum of 5.5 Å.

## 3. Discussion

We compared the position of a Pc molecule in a complex with Cyt *f* in various structures obtained by sequential use of the BD and MD computational methods, the AI method AlphaFold 3, and the Pc–Cyt *f* complex structures determined by paramagnetic NMR and restrained rigid-body MD. In addition, for each structure, we analyzed its compliance to the electron density determined by cryo-EM.

All studied structures are largely similar to each other. The Pc molecule is located near the heme of Cyt *f* and more or less overlaps with the cryo-EM Pc electron density. Moreover, in all considered structures except the central one obtained by cluster analysis of electrostatic encounters in BD calculations, Pc is oriented such that the copper atom is in close proximity to the Cyt *f* heme.

The encounter complex is different from all other complexes, since this state is preliminary to the formation of the functionally active complex. The encounter complex includes many structures that are quite different from each other. In these structures, the position of the Pc molecule is determined only by electrostatic interactions. At this stage of complex formation, the solvent molecules have not yet been displaced from the protein–protein interface. To simulate this step, we used the BD method, which utilized only Brownian diffusion and electrostatic interactions. The structure shown in Figure 4a is the central structure of the encounter complex, in which the Cu–Fe distance is still quite large, and the electrostatic interaction with the positively charged loop of the small domain of Cyt *f* plays an important role. Note that the central structure of the complex should not be confused with the complex as a whole.

When water molecules are eliminated from the protein–protein interface, the encounter complex further transforms into the final one, resulting in a state where the distribution of Pc orientations is significantly reduced. This step was simulated by all-atom MD of the encounter complex central structure with explicit solvent. After about 170 ns of evolution, this structure transformed into a rather stable state, well consistent with cryo-EM electron density. In this process, a region of hydrophobic contact is formed near the heme of Cyt *f* and the copper atom of Pc (Figure 3b). The electrostatic contact of Pc with the small domain of Cyt *f* is maintained, but this small domain is slightly shifted towards Pc, thus allowing the formation of a tight hydrophobic contact between the Pc and Cyt *f* regions surrounding the cofactors. In this situation, the position of Pc and the region near the Cyt *f* heme correspond well to the electron density, while the mobile loop of the Cyt *f* small domain extends beyond it.

We should note that Sarewich et al. [13] did not detect any structural change in the Cyt *bf* core subunits upon Pc binding to Cyt *f*. This discrepancy may be related to the fact that in the MD model, we did not consider the entire Cyt *bf* complex, but only its *f* subunit [8]. In the entire Cyt *bf* complex, the interaction of the *f* subunit with the Rieske subunit might constrain the position of the small domain of Cyt *f*, preventing it from shifting toward Pc.

NMR detects the same areas of contact between the Pc and Cyt *f* molecules, as we see in the MD simulation. When determining the structure of the Pc and Cyt *f* protein complex, diamagnetic chemical shift changes and intermolecular pseudocontact shifts were used as input in restrained rigid-body MD calculations [11]. As a result, in the obtained complex structures, both the contacts (contact of the regions surrounding the cofactors and contact of the small domain of Cyt *f* with Pc) are achieved not due to a shift of the small domain of Cyt *f*, but due to the rotation of Pc molecule in relation to its position observed in the BD+MD calculation [8].

Surprisingly, the best match to the Pc electron density was demonstrated by an AlphaFold 3 structure. In this structure, the contacts near protein cofactors are almost the same as in the structure obtained by BD+MD, although the contact of Pc with the small domain of Cyt *f* is weaker. The orientation of Pc in this structure almost completely coincides with its orientation obtained by the BD+MD calculation, and at the same time, is very different from the orientation of Pc predicted on the basis of NMR data. This is even more unexpected given that only NMR structures for the Pc–Cyt *f* complex are available in the PDB database, which was used to train AlphaFold 3.

While the orientation of Pc is generally the same in all the structures predicted by AlphaFold 3, these structures differ mostly in the shift of the Pc molecule as a whole. This can be seen from the fact that the distance between the centers of mass of the Pc molecule in two structures is almost equal to or slightly less than the RMS distance between the Pc C_α_ atoms in the same structures. The PAE values calculated by AlphaFold 3 for a pair of interacting molecules Pc and Cyt *f* (about 3 Å) practically coincide with the RMSD between the predicted structures.

## 4. Materials and Methods

We used structures of spinach Pc–turnip Cyt *f* complex (PDB id 2pcf [11]) and poplar Pc–turnip Cyt *f* complex (PDB id 1tkw [12]) obtained by M.Ubbink group using paramagnetic NMR and restrained rigid-body MD. We also used spinach Cyt *bf* complex structure (PDB id 7qrm) as well as spinach Pc–Cyt *bf* complex electron density (EMDB entry EMD-14123) obtained by A.Osyczka group using cryo-EM [13].

In our previous paper [8], we predicted spinach Pc–turnip Cyt *f* encounter and final complex structures using combined multi-scale BD+MD approach. We used the ProKSim (Protein Kinetics Simulator) software v. 661 [24,25] for BD simulations. Rigid-body BD model was used to simulate the diffusion of proteins and the formation of their encounter complexes in solution. It was based on the mathematical apparatus of the Langevin equation, which describes the translational and rotational motion of proteins under the action of a random Brownian and electrostatic forces in a viscous medium [26]. From the point of view of electrostatics, in the BD model, a protein molecule was represented by a low dielectric area with spatially fixed partial charges. The solvent was considered as a high dielectric area with mobile charges (ions), implicitly described through solution ionic strength. A Poisson–Boltzmann calculation [27] was used to determine the electrostatic potential grid around each molecule, as we have described in detail earlier [24].

Initially, two protein molecules were placed in random positions and orientations in the cubic reaction volume 300 × 300 × 300 Å^3^ with periodic boundary conditions. Thirty thousand BD simulations with various initial positions were run until attractive electrostatic energy reached the predefined threshold 20 kJ/mol (8 kT) and the obtained complex structures were analyzed by a hierarchical density-based clustering algorithm [28]. The central structure of the cluster of ‘productive’ electrostatic complexes (i.e., complexes which can evolve to functionally active ones) obtained by BD in our previous paper [8] is presented in the Supplementary Materials. We consider the central structure as the structure having the minimum average RMSD from all the other structures in the set.

In our previous paper [8], we also studied the evolution of this central structure into the final complex using all-atom MD with explicit solvent. A three-dimensional dodecahedron reaction volume with periodic boundary conditions was used. The size of the virtual cell was chosen in such a way that the distance from the protein surface to the nearest box boundary was not initially less than 20 Å. The ionic strength of the solution was set at 100 mM by adding Na^+^ and Cl^−^ ions, and the total charge of the system was zero. Calculations were performed in GROMACS 5.1.4 software [29] with CHARMM27 force field [30,31]. Changes in the electrostatic and hydrophobic contacts along MD trajectory were analyzed and described in paper [21]. The frames from 162 ns till 919 ns we consider as final complex states with Cu–Fe distance around 15 Å (Figure 3b). We extracted the frames from the MD trajectory every 1 ns and spatially aligned them to Cyt *f* (chain C of 7qrm) atoms surrounding heme (see Supplementary Files). The central structure of the final complexes was obtained using gmx cluster of Gromacs [29] and is presented in the Supplementary Materials.

For AI-based prediction of intermolecular complex structure of the proteins Pc and Cyt *f*, we used AlphaFold 3 tool by Google DeepMind (https://alphafoldserver.com/ accessed on 9 October 2024) [22]. We used spinach Pc sequence (UniProtKB accession: P00289, positions 70–168), poplar Pc sequence (UniProtKB accession: P00299, positions 70–168), turnip Cyt *f* sequence (UniProtKB accession: P36438, positions 36–285), and spinach Cyt *f* sequence (UniProtKB accession: P16013, positions 36–285). Cofactors Cu^2+^ ion and Heme C were added.

For AlphaFold 3 prediction of spinach Pc–Cyt *bf* complex, we used the same sequence for spinach Pc (UniProtKB accession: P00289, positions 70–168) and sequences of all resolved protein parts of Cyt *bf* subunits from PDB structure 7qrm. The sequences were obtained by pdb_seq utility of the PDBtools open source software Version 3 (https://github.com/harmslab/pdbtools accessed on 9 October 2024). Two chlorophyll *a* molecules, four hemes B, four hemes C, and a Cu^2+^ ion were added.

For various scores generated by AlphaFold 3, such as pTM, ipTM, and PAE, please refer to the original papers [22,32].

We aligned protein sequences using the Clustal Omega program provided by UniProt (https://www.uniprot.org/align accessed on 9 October 2024) [33].

For visualization of protein structures and electron density spatial distribution, we used the PyMOL 2.5 Molecular Graphics System [34]. All the studied Pc–Cyt *f* and Pc–Cyt *bf* complex structures were spatially aligned to the 7qrm chain C (Cyt *f* located near Pc electron density). To compare Pc positions in Pc–Cyt *f* and Pc–Cyt *bf* complex structures, we calculated distances between Pc centers of mass and RMS distances between Pc C_α_ atoms for every pair of structures spatially aligned as mentioned above.

In this paper, we evaluate how well the spatial position of a protein molecule as a whole corresponds to the spatial position of its experimentally determined electron density. In the original electron density map (EMDB entry EMD-14123 [13]), the voxel size is 0.86 × 0.86 × 0.86 Å^3^. In order to ensure that small changes in the individual atom spatial positions do not affect the result of such evaluation, we smoothed out spatial fluctuations of the electron density by applying moving average to the electron density grid with window size of five cells in each direction (totally 125 neighboring cells, 5 × 5 × 5 cube). Then, we used the resulting smoothed electron density value (in the same units as in the original map) in the location of any individual atom of the protein as a measure of compliance of this atom position to protein electron density and called it the EDC score for this particular atom. To calculate the EDC score for the whole protein, we averaged the EDC score values over all C_α_ atoms.

We used matplotlib Python package [35] to plot the curves in Figure 3b. Data from MD trajectory were sampled every 1 ns. The Cu–Fe distance and numbers of amino acid contacts were taken from our paper [21]. We used moving average with window size of 20 ns to smooth fast fluctuations of the curves.

## 5. Conclusions

Over the past decade, available computing power has increased manyfold thanks to the emergence of hybrid computing architectures and the widespread use of GPUs. This contributed to the rapid development of computer methods for studying the formation of protein–protein complexes and predicting their structure. Both simulation methods and AI-based approaches have developed tremendously. In this work, we applied several of these methods to determine the structure of the Pc–Cyt *f* protein complex and showed that obtained theoretical results are in agreement with experimental data. We believe that such approaches will be useful for studying other transient protein–protein complexes that have not yet been amenable to experimental study.

## Figures and Tables

**Table 1:** Comparison of Pc–Cyt *f* complex structures shown in Figure 4. The names of columns and rows correspond to the panels in Figure 4. (a) Central structure of encounter complexes cluster obtained by BD for spinach Pc and turnip Cyt *f*. (b) Final complex obtained by all-atom MD, initiated from the structure in panel (a). (c) Complex of spinach Pc and turnip Cyt *f* predicted by AlphaFold 3 with the highest ipTM score. (d) Complex of spinach Pc and turnip Cyt *f* predicted by AlphaFold 3 with the lowest ipTM and the highest EDC scores. (e) Complex of spinach Pc and spinach Cyt *f* predicted by AlphaFold 3 with the lowest ipTM and the highest EDC scores. (f) Complex of spinach Pc and spinach Cyt *bf* predicted by AlphaFold 3 with the highest ipTM and the highest EDC scores. (g) NMR structure (PDB id 2pcf, model 9) for spinach Pc and turnip Cyt *f*. (h) NMR structure (PDB id 1tkw, model 7) for poplar Pc and turnip Cyt *f*. Outlined diagonal values stand for Cu–Fe distance|EDC score; upper triangle values on a grey background stand for distances between Pc centers of mass in two structures; lower triangle values stand for RMS distances between Pc C_α_ atoms in two structures. All distances are presented in Angstroms.

Structure	a	b	c	d	e	f	g	h
a	28.4|0.026	17.5	15.5	15.9	15.9	14.4	15.3	19.3
b	21.2	14.3|0.056	2.7	2.2	2.0	3.2	7.0	7.9
c	20.5	4.2	13.3|0.055	1.9	1.3	1.9	7.2	9.5
d	20.7	3.3	2.4	12.6|0.059	0.7	1.6	5.5	7.6
e	20.6	3.1	2.0	0.9	12.8|0.058	1.5	6.2	8.3
f	19.0	3.9	2.4	2.3	2.4	13.9|0.059	5.7	8.3
g	19.4	7.7	9.2	7.1	7.7	7.4	11.4|0.052	4.5
h	23.1	9.5	11.4	9.7	10.3	9.9	6.6	14.2|0.046

## Data Availability

Data are contained within the article or Supplementary Material.

## Supplementary Materials

The following supporting information can be downloaded at: https://www.mdpi.com/article/10.3390/ijms252011083/s1.
