# Supplementary material for: Plastocyanin and Cytochrome f Complex Structures Obtained by NMR, Molecular Dynamics, and AlphaFold 3 Methods Compared to Cryo-EM Data
Source: Int J Mol Sci. 2024 Oct 15;25(20):11083. doi: 10.3390/ijms252011083 (PMC11507376; doi:10.3390/ijms252011083)
Supplement: Supplementary file 1 [file ijms-25-11083-s001.zip › Supplementary/Pc align results | UniProt.pdf]

Align results

Highlight properties View: Overview Wrapped

1tkwA_SEQRES	IDVLLGADDGSLAFVPSEFSISPGEKIVFKNNAGFPHNIVFDEDSIPSGVDASKISMSEEDLLNAKGETFEVALSN	76
2pcfA_SEQRES	VEVLLGGGDGSLAFLPGDFSVASGEEIVFKNNAGFPHNVVFDEDEIPSGVDAAKISMSEEDLLNAPGETYKVTLTE	76

1tkwA_SEQRES	KGEYSFYCSPHQGAGMVGKVTVN	99
2pcfA_SEQRES	KGTYKFYCSPHQGAGMVGKVTVN	99

Feedback Help
