# Supplementary material for: Plastocyanin and Cytochrome f Complex Structures Obtained by NMR, Molecular Dynamics, and AlphaFold 3 Methods Compared to Cryo-EM Data
Source: Int J Mol Sci. 2024 Oct 15;25(20):11083. doi: 10.3390/ijms252011083 (PMC11507376; doi:10.3390/ijms252011083)
Supplement: Supplementary file 1 [file ijms-25-11083-s001.zip › Supplementary/Cyt f align results | UniProt.pdf]

UniProt BLAST Align Peptide search ID mapping SPARQL Tool results Advanced | List Search Help

Align results

Overview Trees Percent Identity Matrix Text Output Input Parameters API Request

Tools Download Add Resubmit

Highlight properties View: Overview Wrapped

```
7qrmC_SEQRES YPIFAQQGYENPREATGRIVCANCHLANKPVDIEVPQAVLPDTVFEAVVRIPYDMQLKQVLANGKKGGLNVGAVL 75
2pcfB_SEQRES YPIFAQQNYENPREATGRIVCANCHLASKPVDIEVPQAVLPDTVFEAVVKIPYDMQLKQVLANGKKGALNVGAVL 75
1tkwB_SEQRES YPIFAQQNYENPREATGRIVCANCHLASKPVDIEVPQAVLPDTVFEAVVKIPYDMQLKQVLANGKKGALNVGAVL 75

7qrmC_SEQRES ILPEGFELAPPDRISPEMKEKMGNLSFQSYRPNKQNILVIGPVPGQKYSEITFPILAPDPATKKDVHFLKYPIYV 150
2pcfB_SEQRES ILPEGFELAPPDRISPEMKEKIGNLSFQNYRPNKKNILVIGPVPGQKYSEITFPILAPDPATNKDVHFLKYPIYV 150
1tkwB_SEQRES ILPEGFELAPPDRISPEMKEKIGNLSFQNYRPNKKNILVIGPVPGQKYSEITFPILAPDPATNKDVHFLKYPIYV 150

7qrmC_SEQRES GGNRGRGQIYPDGSKSNNTVYNSTATGIVKKIVRKEKGGYEINIADASDGREVVDIIPRGPELLVSEGESIKLDQ 225
2pcfB_SEQRES GGNRGRGQIYPDGSKSNNTVYNATAGGIISKILRKEKGGYEITIVDASNERQVIDIIPRGLELLVSEGESIKLDQ 225
1tkwB_SEQRES GGNRGRGQIYPDGSKSNNTVYNATAGGIISKILRKEKGGYEITIVDASNERQVIDIIPRGLELLVSEGESIKLDQ 225

7qrmC_SEQRES PLTSNPNVGGFGQGDAEVVLQDPLRIQGLLFFFASVILAQIFLVLKKKQFEKVQLSEMNF 285
2pcfB_SEQRES PLTSNPNVGGFGQGDAEIVLQDPLR----------------------------------- 250
1tkwB_SEQRES PLTSNPNVGGFGQGDAEIVLQDPLRVQ--------------------------------- 252
```

Feedback

Help
